# Discovering novel disease comorbidities using electronic medical records

**DOI:** 10.1371/journal.pone.0225495

**Published:** 2019-11-27

**Authors:** Shikha Chaganti, Valerie F. Welty, Warren Taylor, Kimberly Albert, Michelle D. Failla, Carissa Cascio, Seth Smith, Louise Mawn, Susan M. Resnick, Lori L. Beason-Held, Francesca Bagnato, Thomas Lasko, Jeffrey D. Blume, Bennett A. Landman

**Affiliations:** 1 Department of Electrical Engineering and Computer Science, Vanderbilt University, Nashville, Tennessee, United States of America; 2 Department of Biostatistics, Vanderbilt University, Nashville, Tennessee, United States of America; 3 Department of Psychiatry & Behavioral Sciences, Vanderbilt University Medical Center, Nashville, Tennessee, United States of America; 4 Department of Radiology and Radiological Sciences, Vanderbilt University Medical Center, Nashville, Tennessee, United States of America; 5 Department of Ophthalmology and Visual Sciences, Vanderbilt University Medical Center, Nashville, Tennessee, United States of America; 6 Laboratory of Behavioral Neuroscience, National Institute on Aging, Baltimore, Maryland, United States of America; 7 Department of Neurology, Vanderbilt University Medical Center, Nashville, Tennessee, United States of America; 8 Department of Biomedical Informatics, Vanderbilt University Medical Center, Nashville, Tennessee, United States of America; University of Toronto, CANADA

## Abstract

Increasing reliance on electronic medical records at large medical centers provides unique opportunities to perform population level analyses exploring disease progression and etiology. The massive accumulation of diagnostic, procedure, and laboratory codes in one place has enabled the exploration of co-occurring conditions, their risk factors, and potential prognostic factors. While most of the readily identifiable associations in medical records are (now) well known to the scientific community, there is no doubt many more relationships are still to be uncovered in EMR data. In this paper, we introduce a *novel finding index* to help with that task. This new index uses data mined from real-time PubMed abstracts to indicate the extent to which empirically discovered associations are already known (i.e., present in the scientific literature). Our methods leverage *second-generation p*-values, which better identify associations that are truly clinically meaningful. We illustrate our new method with three examples: Autism Spectrum Disorder, Alzheimer’s Disease, and Optic Neuritis. Our results demonstrate wide utility for identifying new associations in EMR data that have the highest priority among the complex web of correlations and causalities. Data scientists and clinicians can work together more effectively to discover novel associations that are both empirically reliable and clinically understudied.

## Introduction

Electronic medical record (EMR) systems have been increasingly leveraged for clinical and medical research[1–6]. EMR data provides large sample sizes and information on a wide range of conditions that impact broad populations. EMR systems contain a rich variety of data including lab results, medications, clinical notes, administrative and billing codes, and images. In this work, we introduce a tool, PheDAS (Phenome-Disease Association Study), to perform association studies and identify disease comorbidities across time in EMR data.

Association studies are typically designed to learn the strength of association between one fixed variable and a large set of potentially correlated variables. An early example was the genome-wide association study (GWAS), which identifies genetic variants associated with a specified phenotypic condition[7]. The phenome-wide association study (PheWAS)[8] reverses the direction to compute the association between many phenotypic conditions and a specific genetic variant. Other studies adopt the design to identify associations between non-genetic variables based on information extracted from EMR. For example, comorbidities of Non-Hodgkin’s Lymphoma were found by computing its associations with a set of potential comorbidities extracted from a Medicare claims database[9]. A disease-wide comorbidity map similar to the molecular concept map (MCM) was estimated using an association study design with data extracted from an EMR[10]. Holmes et al used a combination of discharge summaries, diagnostic codes, PubMed database, and Wikipedia articles to identify co-morbidities in three rare diseases[11].

Two of the most important challenges in the design and analysis of association studies are identifying which of the statistical significant results have clinical relevance[12]. An obstacle for traditional statistical methods in these large observational studies is the problem of multiple testing. Because a large number of associations are examined at the same time, the probability of making at least one false claim of significance (the “family-wise error rate”) grows with the number of examinations. Previous association studies have employed traditional multiple hypothesis corrections to control either the family-wise error rate or the false discovery rate, such as Bonferroni or Benjamini-Hochberg p-value adjustments[13,14]. Despite these efforts, association studies have suffered from the problem of reproducibility[15]. GWAS studies are usually followed by meta analyses and replication studies to identify truly significant results[16].

Establishing the clinical relevance of results is an important step that is too often missed or done inadequately in high throughput contexts. The most widely used statistical methods ignore clinical relevance; traditional statistical procedures routinely flag significant results that have no practical meaning. When performing inference on such a large scale, it is not feasible to manually sift through all the estimated effects to determine which of the significant results are most clinically important. Besides, this determination ought not to be made after looking at the results. The most common practice is to focus all or most of the attention on the subset of significant results with the smallest *p*-values. However, findings with the smallest *p*-values have no guarantee of being the most impactful results, and potential important discoveries are often overlooked. What is desired is a principled way to incorporate clinical relevance into the ranking of important findings.

With the aim of directly addressing these challenges, we used second-generation *p*-values (*p*_*δ*_) recently defined by Blume et al to identify associations that are both statistically and clinically significant[17]. Under this approach, we specify *a priori* a null interval hypothesis for effect sizes that are scientifically uninteresting, and only consider as positive findings those associations for which the estimated effect size confidence interval lies completely beyond this null region. Using the second-generation *p*-value as the metric for defining significant results reduces the type I error and false discovery rates as compared to classical point null hypothesis significance tests which use the *p*-value[17]. Specifically, incorporating the null interval forces the type I error to zero as the sample size approaches infinity, thereby proactively adjusting the Type I error to control false discoveries. The subsequent finding are more likely to replicate, almost by definition, and are guaranteed to be clinically relevant.

Exploratory association studies provide an additional third challenge because they can find many associations that are well known, in addition to the potentially surprising results. While the well-known associations can be useful in that they validate the results of the current study and of prior studies, the more important/interesting results are those previously unknown associations which could potentially be used to develop new hypotheses. For external validation of results, previous association studies have used meta analyses,[16] and manual review by experts[11]. Currently there are no methods to identify which associations are empirically reliable but clinically unknown or understudied. In addition, there are no quantitative measures to identify the extent of clinical novelty of these associations. Instead they have to be reviewed on a case-by-case basis which would require a large group of clinical specialists across many areas of expertise and may be more subjective. In this work, we introduce the Novelty Finding Index (NFI), which addresses this challenge and allows for the creation of a tool for mining disease comorbidities that are clinically relevant and can be ranked by novelty (i.e., newness).

## Methods

### Data

#### Study 1: Autism Spectrum Disorder

The data for this study, including demographic and ICD-9 codes, was collected at the Vanderbilt University Synthetic Derivative under IRB approval, in a de-identified form. The index disease group for this study was defined by patients with a diagnosis of ASD (ICD-9 codes– 299.*). The control group is defined by subjects with typical development.

#### Study 2: Alzheimer’s disease

The data for this study was collected through the Baltimore Longitudinal Study of Aging (BLSA), a study that collects longitudinal data of an aging population in order to examine changes in the brain as a person ages[18]. The data contains self-reported ICD-9 codes, along with medical records and demographic data. In this study, the index disease group is defined by individuals who were diagnosed with Alzheimer’s disease through a clinical consensus. The control group for this study is individuals in BLSA who had no cognitive impairment.

#### Study 3: Optic neuritis

The data for this study was collected from Vanderbilt University’s Synthetic Derivative under IRB approval. It contains EMR data including ICD-9 codes and demographic information. The index disease group is defined by patients with codes 377.30–377.39. The control group for this study are subjects with other disorders of the optic nerve or subjects with hearing loss.

### Phenome-disease association study

We developed a python tool to perform phenome-disease association studies (PheDAS). PheDAS is used to identify clinical phenotypes that are associated with a given index disease. A clinical phenotype or a phecode is a code based on hierarchical categorization of ICD-9 (International Classification of Disease—9) codes, which describes a diagnostic “phenotype” by grouping a set of related ICD-9 codes. The ICD-9 codes are coded labels used in billing that describe the relevance of a visit to a particular cluster of symptoms. The visits and procedures themselves are often coded through a separate index known as the Current Procedural Terminology (CPT) system. ICD-9 (or updated -10) codes are associated with each visit or patient history and are necessary for billing to ensure that CPT are codes applicable for each patient. The ~15,000 ICD-9 codes are mapped to 1,865 phecodes as described by Denny et al[19]. For example, “depression” phecode 296.2 groups the ICD-9 codes of “major depressive disorder, single episode, mild degree” (ICD-9 = 296.21), “major depressive disorder, recurrent episode, mild degree” (ICD-9 = 292.31), and “depressive disorder NEC” (ICD-9 = 311). For each phecode, a set of exclusion codes are also defined which can be used to select a control cohort.

**Fig 1:**
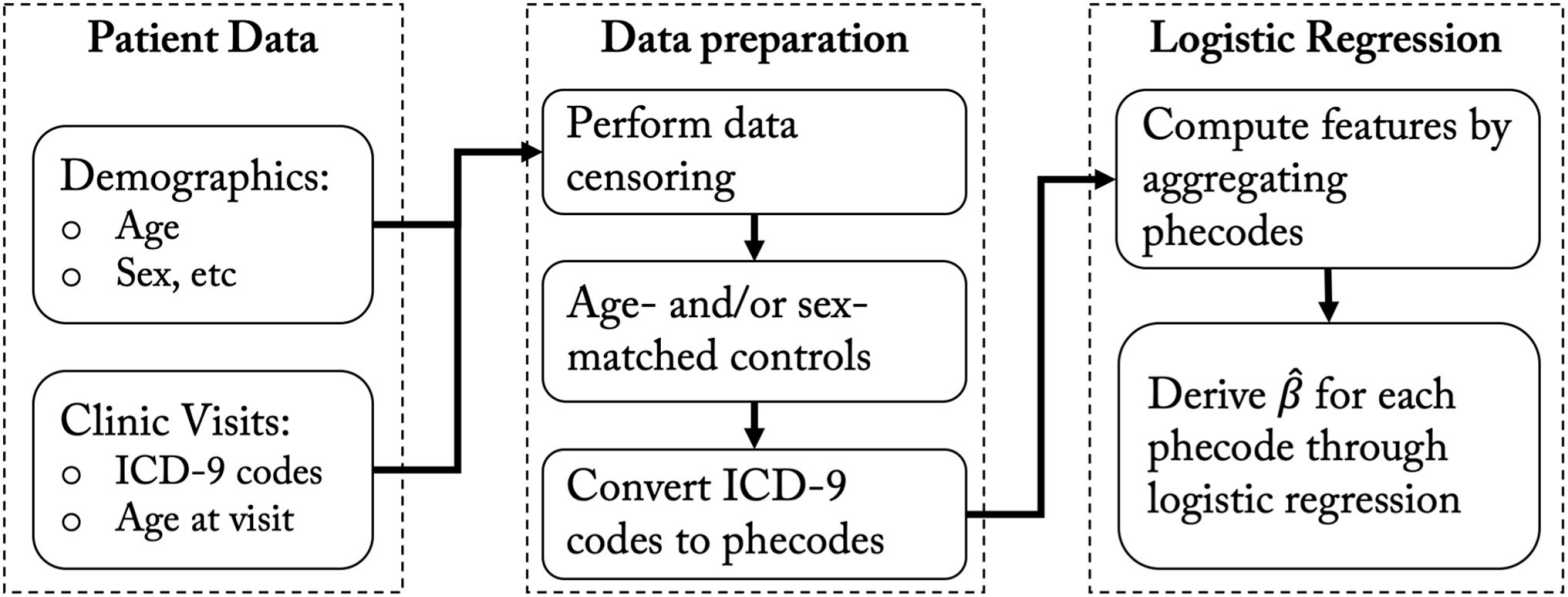
Flow chart for phenome-disease association study. The input patient data required for this analysis is demographic data and clinic visits data. The data is prepared by performing data censoring and control matching based on the experimental design. Next, the ICD-9 codes are converted to phecodes. Finally, logistic regression is performed for each phecode based on aggregate measures and demographic features as described in sections below. To provide a concrete example, consider the Phecode for Alzheimer’s Disease (Phecode: 290.11), which references the ICD-9 code 331.0. ICD-9 331.0 maps to CUI C0002395 and then to 41 UMLS strings. Three example strings are “alzheimer’s disease”, “alzheimers disease”, and “senile dementia”.

Given a disease group and a control group, the PheDAS tool performs a set of logistic regressions to identify significant phenotypes associated with the disease. A flowchart of the process is shown in Fig 1. The ICD-9 codes for each clinical visit and other demographic information are extracted from each subject’s electronic medical record (EMR). Optionally, the time interval for extraction of ICD-9 codes can also be adjusted according to the study design. This can be done by,

- Censoring by age-interval: Selecting an age range within which to perform the analysis. (Ex. In study 1, we analyze the differences between ASD and control population after age 7); or
- Left-censoring with respect to diagnosis: Selecting a time interval prior to year of diagnosis. (Ex. In study 2, we analyze the differences between Alzheimer’s and control population 0–5 years before the diagnosis of the disease); or
- Right-censoring with respect to diagnosis: Selecting a time interval post the year of diagnosis. (Ex. In study 3, we analyze the differences between Optic Neuritis and control population 0–5 years after the diagnosis of the disease).

After defining the interval of the study, ICD-9 codes are extracted for all clinic visits during the period and converted to phecodes using the mapping provided by Denny et al. These codes are denoted by *C* = {*c*_*k*_|*k* = 1…1,865}. For each code *c*_*k*_, an aggregate measure *m*_*k*_ is computed in order to perform the logistic regression. The regression tool can be set to one of the following options:

- Binary measure: aggregate codes to indicate the presence or absence of the phenotype *c*_*k*_(*m*_*k*_ = 0 or 1) in the subject’s record in the given time interval,
- Count measure: aggregate codes to indicate the number of times *c*_*k*_ was present in a subject’s EMR (*m*_*k*_ = *n*), or
- Duration measure: aggregate codes to indicate the time interval between the first and the last time the phenotype *c*_*k*_ was recorded in a subject’s EMR (*m*_*k*_ = *t*).

Additional covariates such as age and sex can also be provided, if available. For each *c*_*k*_, logistic regression is performed based on the following mean model,

$$logit(p(class=disease|c_{k}))=\beta_{0}+\beta_{m}m_{k}+\beta_{a}a_{k}+\beta_{s}s_{k},$$

where *a*_*k*_ is age and *s*_*k*_ is sex. The coefficient of the aggregate measure *β*_*m*_ is used to determine the significance of the association between the disease and phenotype *c*_*k*_. In describing the statistical methods, we will denote *β*_*m*_ by *θ* to allow for some generality, as the methods apply to any parameter of interest in the above regression model. Let the point estimate of *β*_*m*_ = *θ* be denoted by $\hat{\theta }$.

#### Second-generation p-value

We used the second-generation p-value (SGPV) measure described by Blume et al[17] to prioritize or rank potential associations. The SGPV framework requires (1) a pre-defined “indifference zone” or null interval hypothesis around the null effect to denote the set of effect magnitudes that would not be clinically meaningful and (2) an uncertainty interval for the observed association, e.g. a confidence interval, likelihood support interval, or credible interval. The SGPV, denoted by *p*_*δ*_, measures the overlap between the data-supported effect sizes (#2) and the interval null (#1). See Blume (2018) for details[17].

The SGPV equals 0 when #2 and #1 do not overlap. In this case the data only support effect sizes in the alternative hypothesis space. We take all cases where the SGPV is zero, *p*_*δ*_ = 0, to be clinically interesting and statistically ‘significant’. In contrast, when *p*_*δ*_ = 1, the data support only effects that are null or nearly null and not of clinical interest. These results would confirm the lack of association. SGPVs between 0 and 1 are treated as inconclusive as the data support both null and alternative hypotheses.

The interpretation of the coefficient *θ* is different depending on whether *m*_*k*_ is a binary measure, a count measure, or a duration measure. The clinically meaningful effects that make up the null interval will therefore be different in each of these cases. Additionally, the null interval may depend on factors like severity of the outcome. For example, a phecode that increases the odds of having non-specific symptoms such as fever or migraine in Optic Neuritis by a factor of 1.1 may be considered not meaningful, whereas a phecode that increases the odds of having musculoskeletal symptoms in Alzheimer’s by a factor of 1.1 could be an important result to consider.

It is of interest to know how reliable SGPV findings are when *p*_*δ*_ = 0 and whether or not the findings are already known in the literature. To address these two important questions, we estimated the positive predictive value (PPV) when the SGPV is zero and developed a “novelty score” by scraping and searching relevant abstracts in PubMed.

#### Positive predictive value

The positive predictive value (which is the complement of the false discovery rate, FDR) was estimated using an empirical Bayes approach. Define the interval null hypothesis as $H_{0}:\theta \in \Theta_{0}=[\theta_{0}^{-},\theta_{0}^{+}]$ with an alternative hypothesis of $H_{1}:\theta \in \Theta_{1}=(-\infty ,\theta_{0}^{-})\cup (\theta_{0}^{+},\infty )$. Assume that we have a point estimate $\hat{\theta }$ of *θ* that is asymptotically Normally distributed with variance ${\hat{V}}_{n}=V/n$, that is, $\hat{\theta }{∼}^{A}N(\theta ,{\hat{V}}_{n})$. The power function for the SGPV is *P*(*p*_*δ*_ = 0|*θ*) and the form is given in Blume et al.

Under certain assumptions, a reasonable approximation for the PPV is the probability that the null hypothesis is true, given that SGPV equals zero. Applying Bayes’ formula, this is 1 − (1 + *P*(*p*_*δ*_ = 0|*H*_1_)⁄*P*(*p*_*δ*_ = 0|*H*_0_) · (1 − *π*_0_)⁄*π*_0_)^−1^ where *π*_0_ = *P*(*H*_0_) is the analysts’ a-priori probability of the null hypothesis before data were collected. We assign probability distributions *f*_0_ and *f*_1_ to the parameter *θ* under the null and alternative hypotheses, respectively. This allows us to estimate *P*(*p*_*δ*_ = 0|*H*_1_) as $1-\tilde{\beta }=\int_{\Theta_{1}}P(p_{\delta }=0|\theta )f_{1}(\theta )d\theta$, which is a weighted average of the power function over the alternative space, and estimate *P*(*p*_*δ*_ = 0|*H*_0_) as $\tilde{\alpha }=\int_{\Theta_{0}}P(p_{\delta }=0|\theta )f_{0}(\theta )d\theta$, which is a weighted average of the power function over the null space. Therefore, the PPV is equal to

$$1-{\left[1+\frac{1-\tilde{\beta }}{\tilde{\alpha }}\frac{1-\pi_{0}}{\pi_{0}}\right]}^{-1},$$

where we set *π*_0_ = 0.5 which is the default non-informative approach. The probability distribution for the null hypothesis was chosen to be a uniform distribution over the null space, that is, $f_{0}∼Unif[\theta_{0}^{-},\theta_{0}^{+}]$. The probability distribution for the alternative hypothesis was chosen to be a uniform distribution over the observed uncertainty interval $\left({\hat{\theta }}_{l},{\hat{\theta }}_{u}\right)$, that is, $f_{0}∼Unif[{\hat{\theta }}_{l},{\hat{\theta }}_{u}]$. Note that *f*_1_ is a function of the observed data and therefore while the form of *f*_1_ is specified a priori, the actual distribution is not.

#### Novelty score and novelty finding index

The ‘novelty score’ is intended to measure the extent to which a finding is well-studied in the literature. We used published abstracts from the PubMed database to construct the ‘novelty score’ as follows: For each index disease, and for each phecode-disease pairing, we obtained the number of published papers in which these are mentioned in the title, abstract, or keywords section.

**Fig 2:**
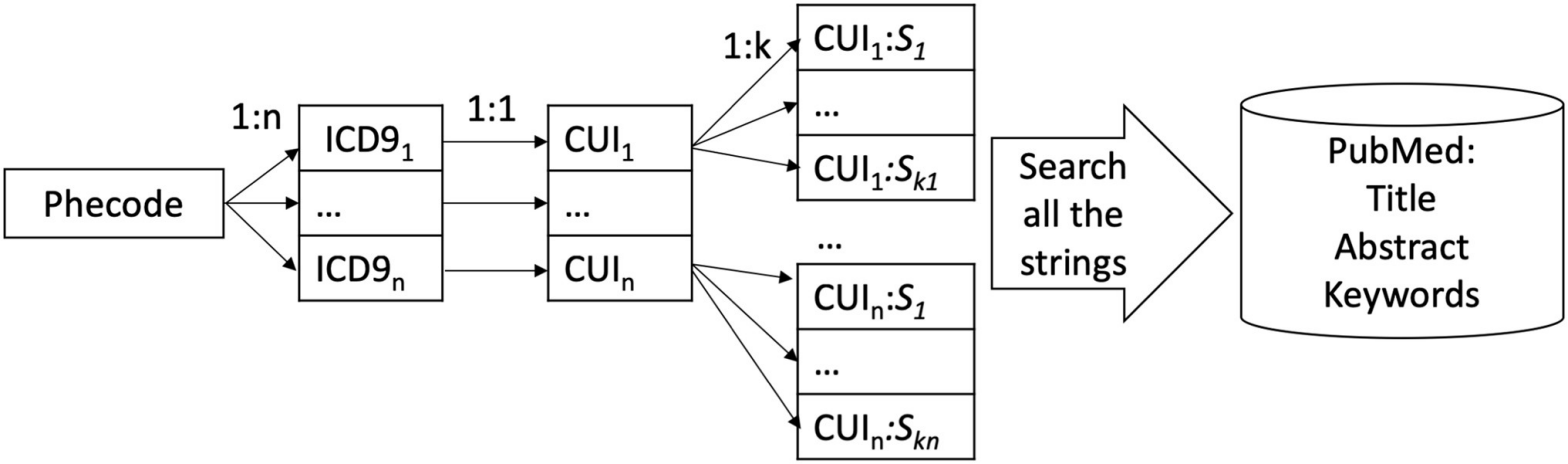
Searching PubMed for associations. For each Phecode, all the ICD-9 codes associated with it are mapped to their CUIs (concept unique identifiers). Next, all the strings associated with the CUIs in UMLS metathesaurus are identified. These strings are used to search all the titles, abstracts and keywords in the PubMed database to identify the counts of academic research papers associated with each phecode.

In order to search PubMed database, we convert phecodes to search strings using the metathesaurus database provided as a part of the unified medical language system (UMLS)[20], as shown in Fig 2. The UMLS metathesaurus defines unique medical concepts that are unchanged over time, identified by the Concept Unique Identifier (CUI). It links strings with the same meaning from over 200 different source vocabularies to the same CUI. ICD-9 codes are included as a part of source vocabularies provided by UMLS. For each phecode, the ICD-9 codes attached to it are linked to a CUI. Next, all possible strings associated with the CUI are extracted from the metathesaurus to be used as search strings. Henceforth, we will take ‘mentioned’ to mean the CUI terms linked to a phecode to be mentioned in either the title, abstract, or keywords section.

We then compute the proportions of published papers that mention the phecode-disease pairing out of all published papers that mention the disease (termed the ‘PubMed proportion’). This proportion measures whether the associations between the outcome and the predictor phecodes are well-studied in the literature. Note that well-studied does not necessarily mean well-known to be associated (i.e., the PubMed proportion should not be interpreted as the estimated probability that an association exists). We denoted the novelty score by $N_{s}=1-\hat{F}(x)$, where $\hat{F}(x)$ is the empirical cumulative distribution function estimated with the PubMed proportions under consideration.

Then, to provide a ranking that accounts for both the reliability of the finding (PPV) and its relative novelty (*N*_*s*_), we define a Novel Finding Index (NFI) as *NFI* = (*PPV* · *N*_*s*_) · 10. The purpose of the scale factor of 10 is to move the *NFI* away from the (0, 1) scale, to prevent misinterpretations of the *NFI* as a probability.

## Results

PheDAS is an EMR-based open-source association study tool that can be used to evaluate relationships between a fixed condition or disease of interest and other clinical phenotypes. We demonstrate the use of this tool in three studies: 1) Autism Spectrum Disorder (ASD) 2) Alzheimer’s Disease, and 3) Optic Neuritis.

**Fig 3:**
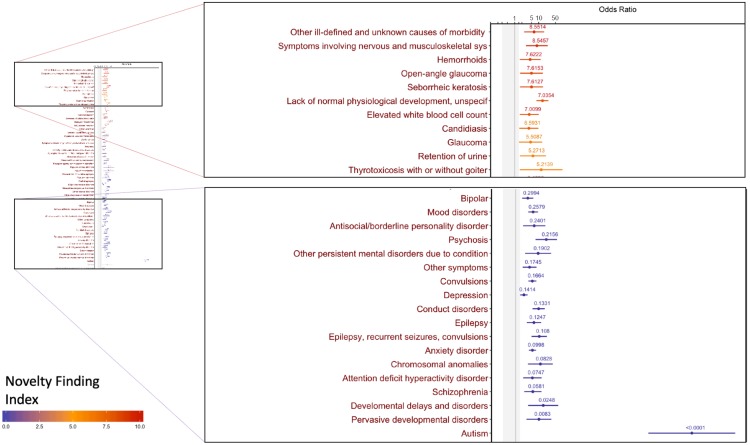
Significant associations for ASD presented as an odds ratio plot. Each dot represents the point estimate of the odds ratio computed from a logistic regression and the line indicates its 95% confidence interval. Each condition is color coded by its novelty finding index, the value of which is displayed above the confidence interval.

**Fig 4:**
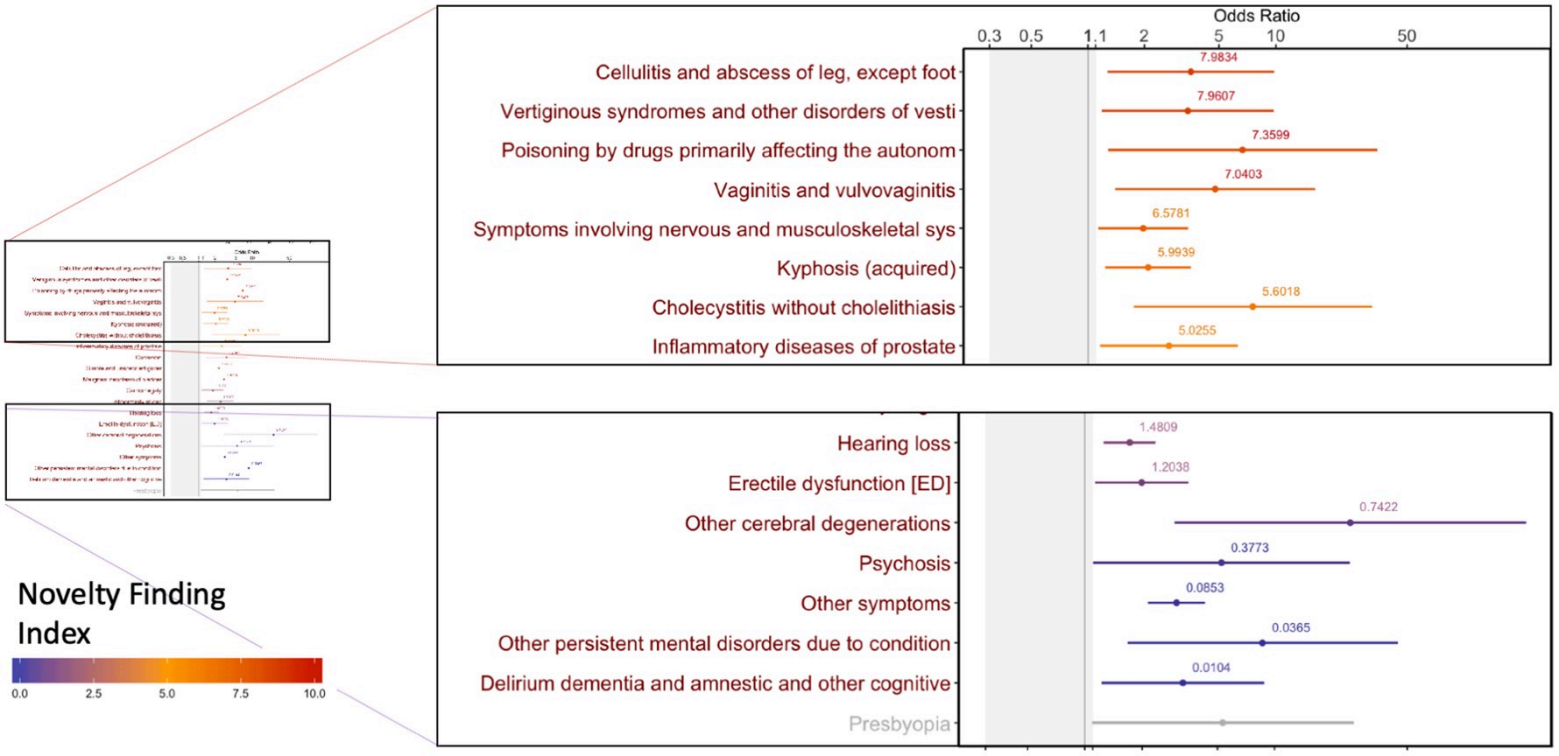
Significant associations 0–5 years before a diagnosis of Alzheimer’s disease presented as an odds ratio plot. Each dot represents the point estimate of the odds ratio computed from a logistic regression and the line indicates its 95% confidence interval. Each condition is color coded by its novelty finding index, the value of which is displayed above the confidence interval.

**Fig 5:**
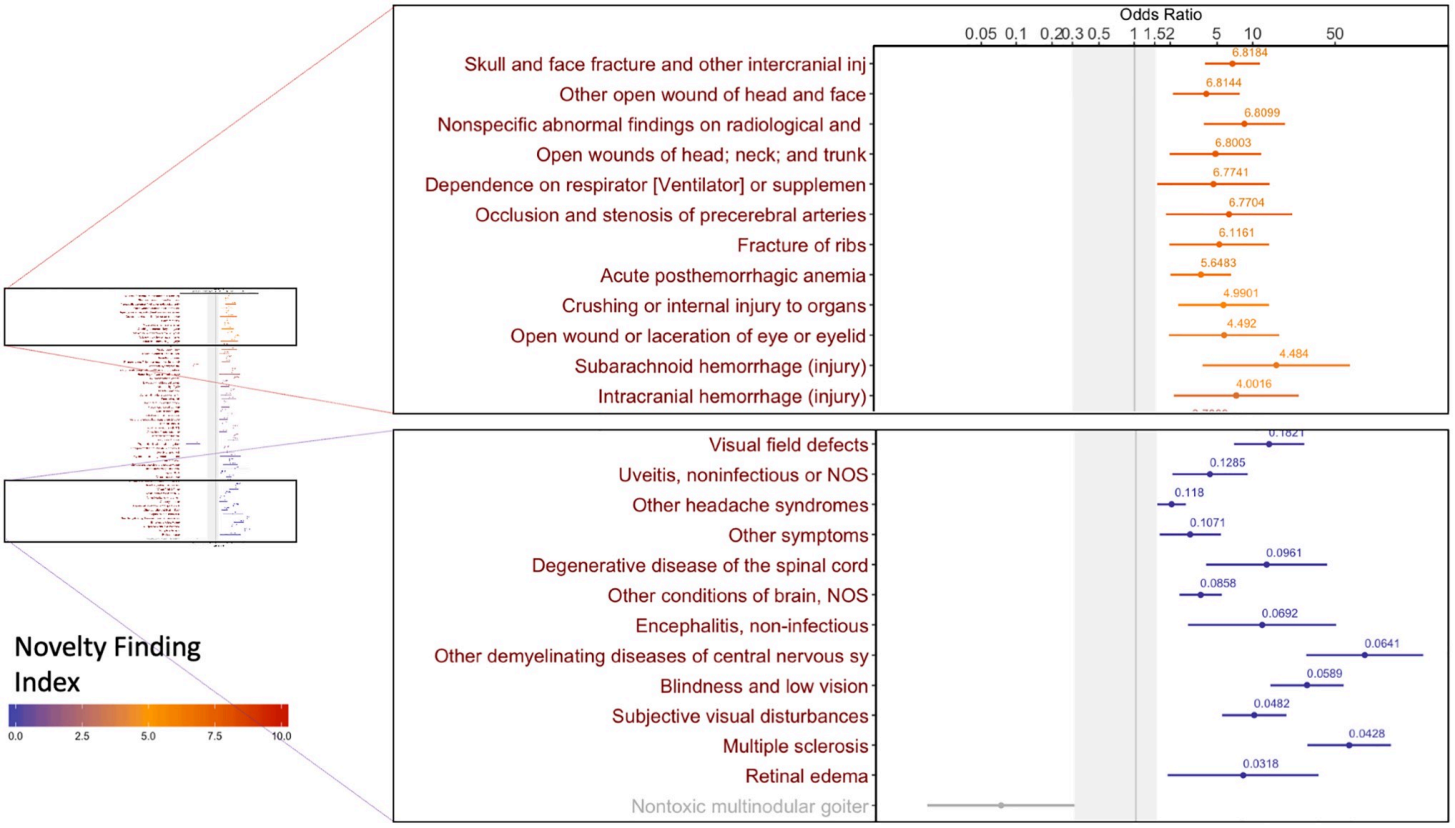
Significant associations 0–5 years after a diagnosis of optic neuritis presented as an odds ratio plot. Each dot represents the point estimate of the odds ratio computed from a logistic regression and the line indicates its 95% confidence interval. Each condition is color coded by its novelty finding index, the value of which is displayed above the confidence interval.

The input to the PheDAS tool is in the form of a list of clinical visits with the recorded ICD-9 codes and age at each visit for each subject, along with the status of the condition of interest (0 = absent, 1 = present). For each experiment, a clinically meaningful null-interval is set prior to the analysis. In Figs 3–5, the null interval is indicated by a gray band. The output provides an odds ratio plot for the point and interval estimates, highlighting significant phecodes that are associated with the condition of interest, color-coded by their Novelty Finding Index (NFI). NFI values near 0 suggest that the finding is well known or likely to be a false positive, while values near 10 suggest a novel and reliable finding (Figs 3–5).

### Study 1: Autism Spectrum Disorder

We first studied co-morbidities that occur in patients with ASD after early childhood. The dataset included records of 1,234 subjects diagnosed with ASD (926 male and 308 female) and 1,234 age-matched controls without ASD (932 male and 302 female). We included Phecodes for all visits at which the patient was at least seven years old. We chose a null interval of [0.3, 1.5] based on clinician input. In practice, it would be ideal to have the intervals be chosen based on consensus of a team of clinical experts to reduce subjectivity. Note that findings in the “negative” direction (decreased odds of ASD when the phecode is present) were decided to be of less clinical interest than findings in the “positive” direction (increased odds of ASD when the phecode). The same will be true in all 3 examples presented. All data were accessed in de-identified form through Vanderbilt’s Synthetic Derivative.

Several strong associations, such as epilepsy, mood disorders, developmental delays, and chromosomal anomalies produced a small NFI, suggesting that these were previously well known (Fig 3).

The novel association of elevated white blood cell count (NFI = 7.01, OR = 4.00, 95% CI 1.62–9.90, SGPV = 0) represents a possible new target to examine a mechanistic theory of ASD. Immune dysregulation has been repeatedly reported in ASD[21] and higher cytokines in response to immune challenge are associated with greater behavioral impairment[22]. Immune dysfunction has clear CNS sequelae, including effects on neurogenesis, synaptic pruning, plasticity, and neuronal function[23]. However, little is known on the mechanistic level about the timing and trajectory of neuro-immune interactions in autism. One theory posits that the role of inflammatory signaling in brain masculinization combined with elevated immune response could explain the high male:female ratio in autism[24]. The potential for addressing these fundamental neurodevelopmental questions using data from electronic health records is stunning, given its longitudinal nature and sample size that allows for the identification of subgroups or the presence of enough females to meaningfully examine sex differences. The novel association detected by PheDAS of elevated leukocytes facilitates more specific hypothesis generation on a topic that has struggled to gain traction in human subjects research. This, for example, could guide future prospective longitudinal studies of neuroimmune interactions in infants at high risk for ASD, moving the field closer to a mechanistic understanding of the impact of immune dysfunction throughout development.

The novel association with glaucoma (NFI = 5.51, OR = 4.54, 95% CI 1.51–13.67, SGPV = 0) may represent another example of how PheDAS may be used. While no current studies have addressed glaucoma in ASD, there is evidence of genetic overlap between glaucoma and ASD[25]. As ASD is a highly heritable disorder, understanding novel genetic associations, especially ones that may be present in unaffected family members can aid early identification and risk assessment for ASD. Additionally, it can highlight the possible common pathways that may represent risk for two different disorders.

### Study 2: Alzheimer’s disease

We examined predictive factors of Alzheimer’s disease by including phecodes of visits between 0 and 5 years prior to the estimated date of diagnosis. The dataset included records of 242 subjects with Alzheimer’s Disease (145 male, 97 female) and 789 age- and sex-matched controls with no dementia diagnosis (499 male, 290 female). Matching was performed based on available data to maximize power. We chose a null interval of [0.3, 1.1] based on clinician input. All data were studied in de-identified form under institutional review board approval.

The well-known associations identified included psychosis, cerebral degenerations, and gait abnormalities (Fig 4). Psychosis and delirium can often be seen in clinical practice and are thought to represent increased risk for neurodegenerative processes including AD.

Novel associations in the five years prior to diagnosis included infections and inflammatory processes across several organ systems. The temporal relationship, wherein the systemic comorbidity precedes clinical diagnosis, supports theories that inflammatory processes and neuroinflammation specifically may contribute to the pathogenesis of AD[26,27]. Peripheral inflammatory markers are elevated early in the AD process [28]and are further associated with cerebrovascular disease[29]. Peripheral elevations in pro-inflammatory cytokines may contribute to neuroinflammation either directly, particularly in situations with compromised blood-brain barrier integrity, or indirectly, through cytokine stimulation of afferent peripheral nerves. Other novel findings include neuromuscular disturbances such as altered vestibular function and increased sensitivity to drugs affecting autonomic function.

### Study 3: Optic neuritis

We examined disease progression in optic neuritis by including phecodes of visits between 0 and 5 years after the estimated date of diagnosis, in a population with no previous multiple sclerosis (MS) phecodes. This dataset included 1,085 subjects with optic neuritis (685 male, 405 female) and 1,085 age- and sex-matched controls without a diagnosis of optic neuritis (685 male, 405 female). We chose a null interval of [0.3, 1.5] based on clinician input. All data were studied in de-identified form under institutional review board approval.

The well-known associations identified included visual field defects, subjective visual disturbances, blindness and low vision (Fig 5). These are expected deteriorations of vision from a diseased optic nerve.

A second category of known associations are neurological conditions such as multiple sclerosis, other demyelinating conditions of the brain and degenerative diseases of the spinal cord. These are in line with previous studies that show that there is a significant risk for future MS in patients who have had optic neuritis[30,31].

Novel associations include several conditions related to traumatic injury, such as skull and face fracture (NFI = 6.82, OR = 6.75, 95% CI 3.95–11.55, SGPV = 0), fracture of ribs (NFI = 6.12, OR = 5.22, 95% CI 1.98–13.75, SGPV = 0), crushing or internal injury to organs (NFI = 4.99, OR = 5.67, 95% CI 2.35–13.71, SGPV = 0) and other open wounds of face and neck (NFI = 6.8, OR = 5.75, 95% CI 1.96–16.83, SGPV = 0), suggesting that the partial loss of vision may contribute to such injuries. Acute loss of visual function is well documented in optic neuritis[32]. While visual field defects are recovered within 4 to 7 weeks, there is a delayed mVEP (multifocal visual evoke potential) in regions where there was a visual field loss[33]. An investigation into the relationship between mVEP and increased risk of falls and injuries in optic neuritis population could help explain the novel associations uncovered by PheDAS. Such an investigation can potentially impact therapeutic recommendations for optic neuritis patients to prevent falls and improve visual cognition, such as regular examinations, the use of walk aids, exercise, and video game play[34,35].

An increased risk of fractures has also been noted in patients with MS owing to disability and low bone density[36], so these may also be secondary associations identified by PheDAS. The increased fractures in the immediate aftermath (0–5 yrs) of an optic neuritis diagnosis suggest that some of the symptoms of MS might appear much earlier than previously thought. A longitudinal examination of patients with a diagnosis of optic neuritis and fractures could reveal new criteria for early prediction of MS. Such a finding can have a substantial impact in management of MS, as early intervention with interferon beta-1b has been shown to delay conversion to clinically definite MS[37,38], reduce the risk for progression of disability[37] and significantly lower lesions on T2-weighted MRI scans[38].

## Discussion

In this paper, we describe a new tool for discovering novel disease co-morbidities from routinely-collected EMR data. The co-morbidities may be selectively specified as preceding, co-occurring with, or following the diagnosis of the condition of interest. Our approach can be used for any condition of interest that is captured by the original data collection.

We address the problem of clinical novelty by ranking findings by prior appearance in the scientific literature. We do this by comparing each phecode-disease finding to the number of papers that can be found on PubMed that mention both conditions as a proportion of the number of papers published on the disease of interest. We define a novelty score, which moves the PubMed proportion, which in some sense is on an absolute scale, onto a relative scale. For example, psychosis is scored low on the novelty score (i.e., not considered to be a novel finding) because it is the 4th most frequent predictor phecode that is studied with Alzheimer’s, despite the fact that it has only been studied in about 1% of the papers that mention Alzheimer’s. A novelty finding index (NFI) is derived from novelty score and the positive predictive value of the association, to indicate the novelty and reliability of the finding.

A limitation of this approach is that we are assuming that if an outcome and predictor phecode are mentioned in the title, abstract, or keywords of the same paper, that an association between them was studied in the paper; this is not necessarily the case. For example, in a paper about Alzheimer’s, psychosis may be noted in the ‘background’ section, but the association between them may not be the topic of the paper. Additionally, papers that study associations among a large number of conditions may not fully list all key terms in the title, abstract, or keywords. These papers would be missed by the proposed approach. However, the fact that the two concepts were discussed in the same paper serves as a reasonable proxy to measure association. As natural language processing (NLP) research advances, it would be interesting to evaluate the of NLP extracts as possible avenues of improving the specificity of patient context[39][40].

NFI has a two-fold benefit. First, the PheDAS methodology can be validated by the novelty finding index by automatically identifying phecode predictors that are well-known by the scientific community. The researcher is assured that the results of the experiment are likely correct, thereby increasing confidence in the analysis. For instance, in our ASD example we see a majority of significant associations that have a low NFI and are well-reported in ASD literature including psychiatric conditions, developmental disorders and seizure disorders. The second advantage, which is the novel aspect of this method is that NFI could be used for hypothesis exploration. It provides a unique and powerful tool to explore empirical relationships in large databases to uncover co-morbidities, risk factors and prognostic factors that were previously under-reported or under-studied. The methodology presented in this paper has the potential to improve understanding of disease etiology and progression and directly impact patient care.
